# The global dataset of historical yields for major crops 1981–2016

**DOI:** 10.1038/s41597-020-0433-7

**Published:** 2020-03-20

**Authors:** Toshichika Iizumi, Toru Sakai

**Affiliations:** 1 National Agriculture and Food Research Organization (NARO), Tsukuba, Japan; 2 Japan International Research Center for Agricultural Sciences (JIRCAS), Tsukuba, Japan

**Keywords:** Environmental sciences, Agroecology, Agriculture

## Abstract

Knowing the historical yield patterns of major commodity crops, including the trends and interannual variability, is crucial for understanding the current status, potential and risks in food production in the face of the growing demand for food and climate change. We updated the global dataset of historical yields for major crops (GDHY), which is a hybrid of agricultural census statistics and satellite remote sensing, to cover the 36-year period from 1981 to 2016, with a spatial resolution of 0.5°. Four major crops were considered: maize, rice, wheat and soybean. The updated version 1.3 was developed and then aligned with the earlier version 1.2 to ensure the continuity of the yield time series. Comparisons with different global yield datasets and published results demonstrate that the GDHY-aligned version v1.2 + v1.3 dataset is a valuable source of information on global yields. The aligned version dataset enables users to employ an increased number of yield samples for their analyses, which ultimately increases the confidence in their findings.

## Abstract (summary)

**Table:** 

Measurement(s)	yield trait
Technology Type(s)	satellite imaging • digital curation • computational modeling technique
Factor Type(s)	year of crop yield data collection
Sample Characteristic - Environment	area of cropland
Sample Characteristic - Location	Earth (planet)

Machine-accessible metadata file describing the reported data: 10.6084/m9.figshare.11903277

## Background & Summary

Crop yield (production per unit harvested area) is an essential variable in many disciplines. Global yield datasets for the historical past have increasingly been used to analyze climate-crop relationships, food production potential, food supply and demand, carbon and nitrogen cycling, greenhouse gas emissions from agriculture and land-use change. Recently, food production losses caused by weather and climate extremes under changing climate and improved stakeholder preparedness are concerns for many societies as the world experiences population growth and subsequent increases in the demand for agricultural products.

An analysis of climate-crop relationships, in particular, the consequences of weather and climate extremes on food production, requires a spatially explicit yield dataset spanning several decades. At the global scale, such a dataset has only recently been developed. The global dataset of historical yield for major crops (GDHY)^1^ is an example of such a dataset. The GDHY is a hybrid of agricultural census statistics and satellite remote sensing. Crop harvested area maps, crop calendar and share of production amount in different growing seasons for a crop are also used as inputs for the GDHY dataset. Therefore, the grid-cell yield values recorded in the GDHY dataset are model estimates rather than observations. Since its development and initial release in November 2013, efforts have focused on improving the data quality, assessing uncertainties and extending the time coverage to include more recent years.

The previous version 1.2 of the GDHY^2,3^ covers the period of 1981–2011. Here, we updated the GDHY dataset to include 2016 (that is, version 1.3) to meet the increasing demand for yield data from the scientific community, food agencies and agrobusinesses. However, the satellite products and reanalysis data used as the inputs for the development of version 1.3 are different from those used in earlier versions, as elaborated later in this article. This difference requires an alignment of version 1.3 with version 1.2 to ensure the continuity of the annual yield time series in the GDHY. Such alignment is essential for many applications in which time series analysis is often utilized, e.g., to depict historical yield patterns and linkages to climate variability and change.

The aligned version v1.2 + v1.3 of the GDHY described in this article^4^ offers yield data for maize, rice, wheat and soybean for the period of 1981–2016, with a spatial resolution of 0.5° and an explicit separation of cropping seasons for some crops (major and second cropping seasons for maize and rice and winter and spring seasons for wheat). The GDHY offers spatially explicit global analyses on crop yields and is especially useful for addressing recent patterns in crop yields and the impacts of recent climate variability and change on global food production; additionally, the GDHY can be used to evaluate global gridded crop model simulations and provide a basis for global and seasonal crop forecasting systems.

## Methods

### Updating the GDHY

The method and procedure used to provide grid-cell yield estimates for version 1.3 of the GDHY are fully described in our related work^1^. In short, the procedure consists of four key steps: (1) the country’s annual yield statistics were obtained from the Food and Agriculture Organization of the United Nations statistical database (FAOSTAT^5^); (2) the grid-cell net primary production (NPP) was calculated using the remotely sensed leaf area index (LAI), the fraction of photosynthetically active radiation (FPAR), reanalysis solar radiation and reported crop-specific radiation-use efficiency to consider the spatial variations in yields within a country; (3) the harvested area map (M3-Crops^6^) and crop calendars circa 2000 (SAGE^7^) were used to address where and when a crop of interest was grown; and (4) when the crop calendars indicated that a crop of interest was harvested twice in a year, the share of production amount by different cropping season of a crop available in the US Department of Agriculture (USDA) report^8^ was used to differentiate the yield estimates for different cropping seasons. Production-weighted mean, instead of arithmetic mean, is utilized when average yield from two cropping seasons with different production share is computed.

Some inputs used in the development of the version 1.3 dataset were different from those used in the version 1.2 dataset (Table 1). The major differences were found in the satellite products and reanalysis data. The LAI and FPAR inputs were changed from the GIMMS3g [Global Inventory Modeling and Mapping Studies third generation products from the AVHRR (Advanced Very High Resolution Radiometer)] products^9^ for the version 1.2, to the more advanced MOD15A2 products^10^ derived from the MODIS (Moderate Resolution Imaging Spectroradiometer) for the version 1.3. The spatial and temporal resolutions of the MOD12A2 products (1-km and 8-day, respectively) were finer than those of the GIMMS3g products (0.083° or 10-km and bi-monthly or 15-day), although the crop harvested area map with a spatial resolution of 10-km was commonly used for both versions 1.2 and 1.3. The daily solar radiation data were also changed from the 1.125°-resolution JRA-25 reanalysis^11^ for the version 1.2 to the 0.563°-resolution JRA-55 reanalysis^12,13^ for the version 1.3.

**Table 1:** A summary of the different GDHY versions considered in this article.

	GDHY		
	Version 1.2	Version 1.3	Aligned version (v1.2 + v1.3)
Reference	Iizumi *et al*.^2,3^	This study	
Time coverage	1981–2011	2000–2016	1981–2016
Spatial coverage	Global (grid-cell yield estimates for some locations are lacking when crop calendars are not available)		
Spatial resolution	0.5°		
Crops	Maize (major/second), soybean, rice (major/second), wheat (winter/spring)		
Country yield statistics	FAOSTAT^5^		
Satellite products	GIMMS3g 0.083° bi-monthly LAI and FPAR^9^	MOD15A2 1-km 8-day LAI and FPAR^10^	Hybrid of dataset versions 1.2 and 1.3
Solar radiation	JRA-25 reanalysis^11^ (1.125° and daily)	JRA-55 reanalysis^12,13^ (0.563° and daily)	
Harvested area	M3-Crops^6^ (0.083° and average around 2000)		
Crop calendar	SAGE^7^ [0.5° (unfilled version) and average around 2000]		
Production share by cropping season	USDA^8^ (national and average in the 1990s)		

More methodological details are available in Iizumi *et al*.^1^.

**Fig. 1:**
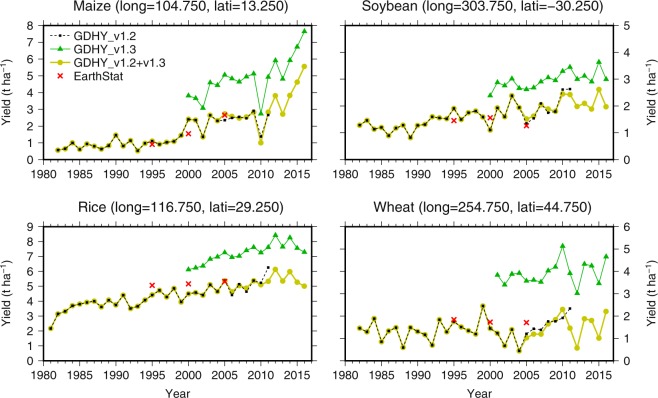
Yield time series in the selected locations for different versions of the GDHY. Yield data obtained from version 1.2, version 1.3 and aligned version v1.2 + v1.3 are presented. Locations indicated by longitude and latitude were arbitrarily selected for explanatory purposes. Five-year average yields at three time points centered on 1995, 2000 and 2005 were obtained from the EarthStat dataset^13^ and are also shown for reference purposes.

The GIMMS3g NDVI (normalized difference vegetation index) used in estimating the GIMMS3g LAI and FPAR were calibrated against the MODIS LAI and FPAR products for the period of 2000–2009 (ref. ^9^). Thus, the continuity of the LAI and FPAR time series at 10-km and 15-day scales was expected. However, the quality-checking of the GDHY version 1.3 dataset revealed persistent discontinuities in annual yield time series between versions 1.2 and 1.3 for some locations, despite the use of the calibrated GIMMS3g LAI and FPAR products (Fig. 1). Yields from the version 1.3 were almost always higher than those from the version 1.2. Addressing the exact reasons for the discontinuities is beyond the scope of this article. However, the different reanalysis solar radiation products between the two versions are one possible reason. And the different spatial resolutions of the satellite products used in versions 1.2 and 1.3 is another possible reason. The version 1.2 dataset uses average NPP over the 10-km grid cell, while the version 1.3 dataset uses the maximum NPP over the 1-km cropland grid cells located within a 10-km grid cell. To solve this problem and supply users a version of the GDHY with continuity, the two versions were aligned, as elaborated in the subsequent section.

### Alignment

The two different versions of the GDHY described above were aligned according to the following procedure. First, in the version 1.2 dataset, the annual yield time series for a given location, crop and cropping season was decomposed into the linear combination of the yield trend component and the yield departure from the trend component:

1 $${Y}_{{\rm{v}}1.2,t}={Y}_{{\rm{v}}1.2,t}+{\acute{Y}}_{{\rm{v}}1.2,t},$$

where ${Y}_{{\rm{v}}1.2,t}$ indicates the annual yield in harvesting year *t* (t ha^−1^); ${Y}_{{\rm{v}}1.2}$ indicates the yield trend component or normal yield (t ha^−1^); and ${\mathop{Y}\limits^{Y}}_{{\rm{v}}1.2}$ indicates the yield anomaly that represents the yield departure from normal yield (t ha^−1^). The normal yield was calculated by applying the 5-year (*t*-4 to *t*) moving average method to the annual time series:

2 $${Y}_{{\rm{v}}1.2,t}=\frac{{\sum }_{t-4}^{t}\,{Y}_{{\rm{v}}1.2,t}}{5}.$$

The yield values in the version 1.3 dataset were also decomposed, as the version 1.2 dataset were processed (${Y}_{{\rm{v}}1.3,t}={Y}_{{\rm{v}}1.3,t}+{\acute{Y}}_{{\rm{v}}1.3,t}$; and ${Y}_{{\rm{v}}1.3,t}=\frac{{\sum }_{t-4}^{t}{Y}_{{\rm{v}}1.3,t}}{5}$).

Second, the two versions of the GDHY were combined into a single time series using the following rule:

3 $${Y}_{{\rm{v}}1.2+{\rm{v}}1.3,t}=\left\{\begin{array}{ll}{Y}_{{\rm{v}}1.2,t} & t=1981,\ldots ,1999\\ {Y}_{{\rm{v}}1.2,t}+\frac{{\mathop{Y}\limits^{{\prime} }}_{{\rm{v}}1.2,t}+{\mathop{Y}\limits^{{\prime} }}_{{\rm{v}}1.3,t}}{2} & t=2000,\ldots ,2010\\ \left[{Y}_{{\rm{v}}1.2,2010}+\left({Y}_{{\rm{v}}1.3,t}-{Y}_{{\rm{v}}1.3,2010}\right)\right]+{\mathop{Y}\limits^{{\prime} }}_{{\rm{v}}1.3} & t=2011,\ldots ,2016\end{array}\right..$$

For the period of 1981–1999, in which only the version 1.2 dataset is available, the yield values in the aligned version (${Y}_{{\rm{v}}1.2+{\rm{v}}1.3}$) are equal to those of version 1.2. For the period of 2000–2010, both versions are available. The normal yields were taken from version 1.2, and the average yield anomalies across the two versions were added to the normal yields. For the remaining period (2011–2016), only version 1.3 is available. The yield anomalies were taken from version 1.3. In contrast, the normal yields were computed by adding the changes in the normal yields between 2010 and the target years (2011–2016), as computed based on version 1.3, to the normal yield in 2010 of version 1.2. When the alignment led to a negative value, the yield value was replaced with zero. By using this procedure, the two versions were harmonized into a single aligned version referred to as the GDHY version v1.2 + v1.3 dataset (Fig. 1).

## Data Records

The GDHY aligned version v1.2 + v1.3 dataset files include the annual crop yield time series in tonnes per hectare (t ha^−1^) for each grid cell. The files are in NetCDF4 format and were generated by using library version 4.6.1.0; they are available at XXXX/yield_YYYY.nc4, where XXXX indicates the crop and cropping seasons (i.e., maize_major, maize_second, rice_major, rice_second, wheat_winter, wheat_spring and soybean); and YYYY indicates the year (i.e., 1981, …, 2016). Only a single cropping season is considered for soybean. The dataset is freely available at PANGAEA^4^.

## Technical Validation

### Approaches for validation

We used two different methods to validate the GDHY aligned version v1.2 + v1.3 dataset: (1) it was compared with another dataset developed by different research group with the authors; and (2) an analysis conducted in earlier peer-reviewed literature was reproduced using the aligned dataset to confirm whether the reproduced results resemble the earlier ones when datasets with different spatial resolutions were analyzed.

### Comparison with another dataset

Another global, spatially explicit, historical yield dataset described in Ray *et al*.^14^ is available at the EarthStat website (http://www.earthstat.org/). We downloaded the dataset labeled “Harvested Area and Yield for 4 Crops (1995–2005)”. In this dataset, the average yield and average harvested area of the four crops at three time points [1995 (1993–1997), 2000 (1998–2002) and 2005 (2003–2007)] are available. Because the original dataset has a grid size of 5 minutes by 5 minutes in longitude and latitude, we aggregated the EarthStat yield data into a grid size of 0.5° by 0.5° in longitude and latitude for a consistent comparison. The average harvested area map at the corresponding time point from the EarthStat was used as the weight when the EarthStat average yield data at a given time point were spatially aggregated.

**Fig. 2:**
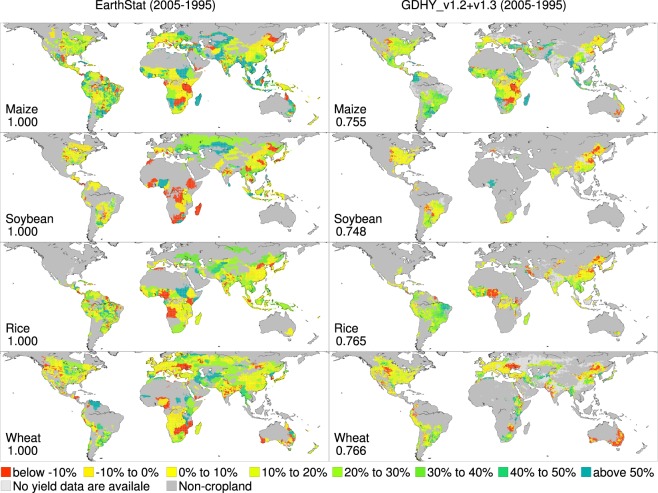
Yield changes for the 1995–2005 period for different datasets. The EarthStat dataset (left) and the GDHY aligned version v1.2 + v1.3 dataset (right) were used to compute the average yield at three points: 1995 (1993–1997), 2000 (1998–2002) and 2005 (2003–2007). Changes in the average yield between 1995 and 2005, relative to 2000, are presented. The numbers shown in each panel indicate the kappa efficient value computed against the EarthStat dataset using the 10 color-coded categorical yield change data over the land area.

For the GDHY aligned version dataset, the average yields for the three time points were computed using the harvested area in 2000 as the weight throughout the study period because no time-varying harvested area map is available for any version of the GDHY. The changes in average yield between 1995 and 2005, relative to 2000, were computed using the two different datasets and are shown in Fig. 2. Annual time series data of the EarthStat dataset are not publicly accessible. Therefore, the two datasets were compared in terms of changes in average yield between the two time points. The calculated yield changes were color-coded according to the 10 categories (the 8 yield change categories from “Below −10%” to “Above 50%”, “No yield data are available” and “Non-cropland” in Fig. 2). Then, the inter-dataset agreement was measured by the kappa coefficient^15^ using the categorical yield change data. The kappa coefficient values ranged from 0.748 to 0.766, indicating good agreement between the EarthStat dataset and the GDHY aligned version dataset. The fact that the EarthStat dataset is solely based on national or subnational agricultural census statistics^14^ underpins the reliability of the GDHY aligned version dataset.

### Reproduction of earlier analysis results

We repeated the analysis described in Iizumi *et al*.^16^ that estimated the impacts of the El Niño Southern Oscillation (ENSO) on global yields. The GDHY version 1.0 dataset^1^ (1.125° and the time coverage of the 25-year period from 1982 to 2006; see Table A in Supporting Information of Iizumi *et al*.^2^ for more details) is used in Iizumi *et al*.^16^. We used the GDHY aligned version dataset for the period of 1981–2016 for reproduction. The reason for the different time periods is that the validation of the aligned version dataset is the main purpose of this reproduction, and the yield data for the period of 1982–2006 in the aligned version are solely based on version 1.2 (see Alignment section in this article). For these reasons, we used the time period of 1981–2016 for the aligned version dataset, with the assumption that the average ENSO impacts on yield is less sensitive to the choice of time period studied. Because of the longer study period than that used in the original work, we replaced the Extended Reconstructed Sea Surface Temperature version 4 (ERSSTv4) dataset^17^ with the ERSSTv5 dataset^18^. Therefore, the method used to address the yield impacts of ENSO is an expanded version of description in our related work^16^.

**Fig. 3:**
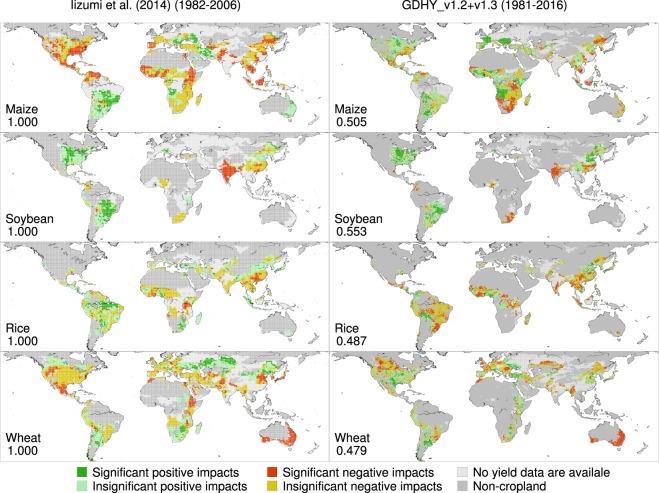
Yield impacts of El Niño for different versions of the GDHY. The original results of Iizumi *et al*.^16^ are based on the GDHY version 1.0 dataset for the period of 1982–2006 (left), while the reproduced results are based on the GDHY aligned version v1.2 + v1.3 dataset for 1981–2016 (right). The numbers shown in each panel indicate the kappa efficient value computed against the original results using the 6 color-coded categorical yield impact data over the land area.

**Fig. 4:**
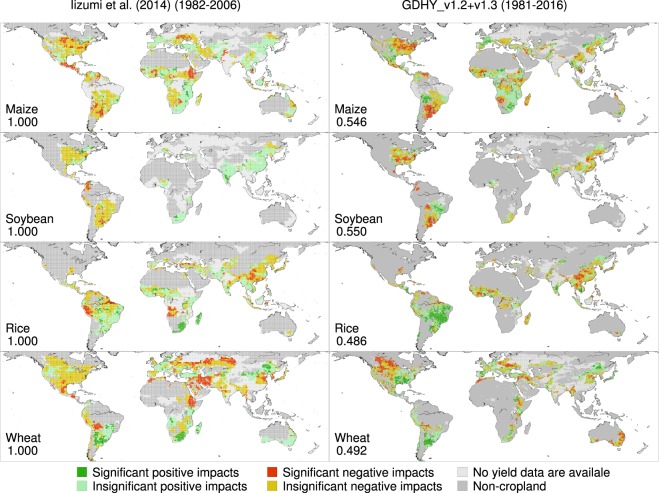
Yield impacts of La Niña for different versions of the GDHY. Same as Fig. 3 but for La Niña.

The kappa coefficient values calculated against the original results (interpolated into 0.5° resolution for a consistent comparison) ranged from 0.487 to 0.553 for the impacts of El Niño, which is a warmer phase of ENSO (Fig. 3). This result indicated an intermediate level of agreement in the 6 color-coded categorical yield impact data between the original and reproduced results. The comparison for the impacts of La Niña, a cooler phase of ENSO, showed a similar level of agreement, as indicated by the kappa coefficient values of 0.486–0.550 (Fig. 4). These agreement levels are reasonable if one considers the difference in spatial resolution and the subsequent difference in spatial coverage across the two versions. The original results have a larger spatial coverage than that of the reproduced results because the larger grid cells (1.125°) used in the version 1.0 dataset often have effective yield values even when yield data for most smaller grid cells (0.5°) located within a larger grid cell are missing.

## Usage Notes

Any versions of the GDHY, including the aligned version v1.2 + v1.3, are a valuable source of information on global yields. However, caution is necessary when the goal is to make user findings derived by analyzing the GDHY robust against the inherent uncertainties in the dataset. The following is a non-exclusive summary of technical notes users should be aware of.

The yields available in the GDHY are model estimates and not free from error due to imperfect modeling, inaccurate inputs, misreporting in agricultural census statistics, and use of time-constant information. Examining the same working hypothesis using other yield datasets (preferably, observed yields) in addition to the GDHY is a good practice to increase the confidence in the findings (e.g., refs. ^2,19,20^).

Different conclusions could be made if different yield datasets were analyzed^2,21^. Practices to avoid leading to conclusions sensitive to the choice of yield dataset are important. Such practice includes utilizing statistics of yield data (e.g., multi-year average yield, relative yield change) or categorical yield data for analysis, as was presented in the Technical Validation section of this article, instead of analyzing raw yield values. Similarly, different spatial resolutions of yield datasets could lead to different conclusions^19,22,23^, and therefore, an examination of a user’s conclusions against uncertainty of this kind is encouraged.

Yields in some locations are lacking in the GDHY. A country or global production total aggregated from grid-cell yields were underestimated if the yield dataset, of which the spatial coverage was incomplete, was analyzed. Calculating a country average yield and then multiplying it by a country’s harvested area are appropriate methods to obtain reasonable estimates of total production for a given spatial unit using the GDHY. Note that the beginning and ending years of the GDHY (i.e., 1981 and 2016, respectively, for the aligned version) have many missing values in the Southern Hemisphere because crop durations in the region often span two calendar years and yields cannot be estimated due to incomplete crop durations.

## Data Availability

The GDHY aligned version v1.2 + v1.3 dataset is produced by combining versions 1.2 and 1.3 using a purpose-build program written in Fortran90 with the standard mathematical library. The program code was compiled on the MacOS platform but is potentially applicable to other platforms (e.g., Windows and UNIX). The code is available from the corresponding author upon request.
